# Akute muskulotendinöse Ruptur des M. pectoralis major: Fallbericht einer seltenen Verletzung und Literaturübersicht

**DOI:** 10.1007/s00113-021-00997-6

**Published:** 2021-04-19

**Authors:** Maximilian Hinz, Benjamin D. Kleim, Felix Mayr, Andreas B. Imhoff, Sebastian Siebenlist

**Affiliations:** Abteilung und Poliklinik für Sportorthopädie, Klinikum rechts der Isar, Technische Universität München, Ismaninger Str. 22, 81675 München, Deutschland

**Keywords:** Sportverletzung, Kraftsport, Rekonstruktion, Bankdrücken, Chronisch, Sports injury, Strength training, Repair, Bench press, Chronic

## Abstract

Die Pectoralis-major-Ruptur (PMR) ist eine seltene Verletzung, die v. a. beim Kraftsport aufritt. Vorgestellt wird der Fall eines 31-jährigen Profibasketballspielers, der sich beim Bankdrücken eine Komplettruptur am muskulotendinösen Übergang des M. pectoralis major (PM) zugezogen hatte. Drei Wochen nach dem erlittenen Trauma erfolgte bei persistierenden Schmerzen und Kraftdefizit die Refixation des PM. Drei Monate postoperativ konnte der Patient bei vollem Bewegungsumfang schmerzfrei in den Basketballsport zurückkehren. Die Verletzungsentität wird vor dem Hintergrund der aktuellen Literatur diskutiert und das operative Vorgehen im Detail dargestellt.

## Abstract (en)

Rupture of the pectoralis major muscle (PMR) is a rare injury that occurs primarily during strength training. The case of a 31-year-old professional basketball player who suffered a rupture of the pectoralis major muscle (PM) at the myotendinous junction while performing bench presses is presented. Due to persistent pain and decreased muscle function, surgery was performed 3 weeks after the initial trauma. The patient returned to playing basketball pain-free with full range of motion 3 months following refixation of the PM. This article discusses the distinct characteristics of this injury in the context of the current literature and the operative approach is presented in detail.

## Einleitung

Die Inzidenz der M.-pectoralis-major-Rupturen (PMR) stieg in den letzten Jahren an, was vermutlich auf eine Popularitätszunahme von Kraftsportarten (z. B. Kraftdreikampf, Bodybuilding, Crossfit) zurückzuführen ist. Die Mehrzahl der PMR tritt bei Männern zwischen dem 20. und 40. Lebensjahr während des Bankdrückens auf, wobei es sich in den meisten Fällen um isolierte Verletzungen des sternokostalen Anteils handelt [1].

Während des Bankdrückens dehnen sich am Ende der exzentrischen Abwärtsbewegung, bei der sich die Oberarme in Abduktion und Extension befinden, die inferioren Muskelfasern des Pars sternocostalis maximal und stellen damit eine mechanische Schwachstelle dar [2].

**Figure 1:**
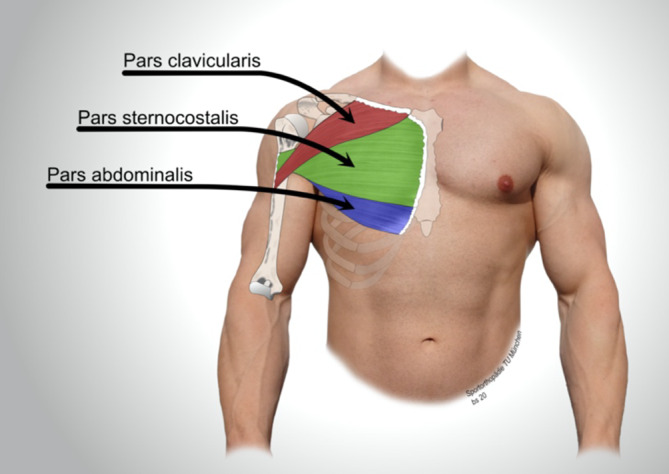

Der M. pectoralis major (PM) besteht aus 3 Anteilen (Abb. 1). Der klavikuläre Anteil (Pars clavicularis) bildet das vordere Sehnenblatt, ist uniform und entspringt am medialen Anteil der Klavikula. Das hintere Sehnenblatt wird aus dem sternokostalen Anteil (Pars sternocostalis) und dem abdominalen Anteil (Pars abdominalis) gebildet. Aufgrund der funktionellen Einheit als hinteres Sehnenblatt werden in der Literatur vermehrt beide Muskelanteile zusammengefasst [3].

Der Pars sternocostalis besteht aus mehreren einzelnen Muskelsträngen, die dem Sternum, dem Knorpel der 1. bis 6. Rippe sowie der Aponeurose des M. obliquus externus abdominis entspringen. Der abdominale Anteil setzt an der 5. bis 6. Rippe sowie der Faszie des M. obliquus externus abdominis und des M. transversus abdominis an [2, 3].

Humeralseitig inserieren alle Anteile gemeinsam lateral der langen Bizepssehne an der sog. Crista tuberculi majoris. Die superioren, klavikulären Fasern inserieren am kaudalen Anteil der Insertion und überkreuzen so die inferioren, sternokostalen und abdominalen Fasern, die weiter kranial ansetzen. Der Sehnenansatz erreicht eine Längsausdehnung von 5–8 cm und eine Dicke von 1–3 mm [2–5]. Rupturen des PM sind am häufigsten im Bereich des humeralen Ansatzes lokalisiert und treten in den allermeisten Fällen am Knochen-Sehnen-Interface auf [6, 7]. Im vorliegenden Fall handelt es sich dagegen um eine muskulotendinöse Ruptur.

## Fallvorstellung

### Anamnese

Ein 31-jähriger professioneller Basketballer verspürte beim Bankdrücken einen plötzlich einschießenden Schmerz in der linken Brust. Zwölf Tage später wurde der Patient aufgrund einer anhaltenden Schwellung der linken Brust sowie eines Kraftdefizits bei Innenrotation und Adduktion der linken Schulter vorstellig.

### Befund

**Figure 2:**
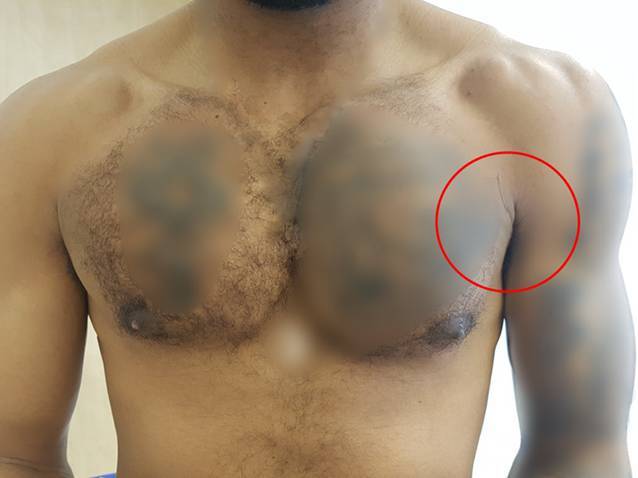

Bei der körperlichen Untersuchung imponierte eine seitendifferente Silhouette der linksseitigen Brustmuskulatur mit einem nach medial retrahierten Muskelbauch des linken PM mit sichtbarem Konturzeichen (Abb. 2). Das „prayer sign“ zeigte sich in der Funktionsuntersuchung positiv [8]. Bei diesem Funktionstest wird der Patient gebeten, die Handflächen vor der Brust aneinanderzudrücken. Diese „Gebetsposition“ („prayer position“) führt zu einer isometrischen Brustmuskelkontraktion und kann eine Asymmetrie der Brustwand und damit eine Ruptur des PM offenbaren. Ein Hämatom war nicht vorhanden (Abb. 2).

**Figure 3:**
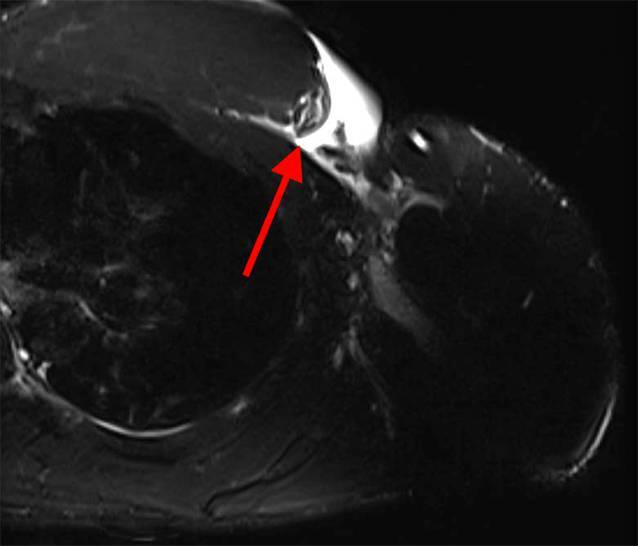

Die MRT-Untersuchung zeigte eine – bis auf nur noch wenige erhaltene Restfasern der Pars abdominalis – komplette Ruptur der Pars clavicularis und Pars sternocostalis des PM am muskulotendinösen Übergang (Abb. 3). Die gemeinsame Sehneninsertion war humeralseitig intakt.

### Diagnose

*Muskulotendinöse Komplettruptur (Typ III-C nach Tietjen *[9]*) der Pars clavicularis und sternocostalis des linksseitigen M. pectoralis major.*

### Therapie und Verlauf

Aufgrund des hohen funktionellen Anspruchs des Patienten wurde die Indikation zur operativen Refixation gestellt. Der Eingriff wurde 19 Tage nach dem Unfallereignis in Allgemeinanästhesie in „Beach-chair“-Lagerung durchgeführt.

**Figure 4:**
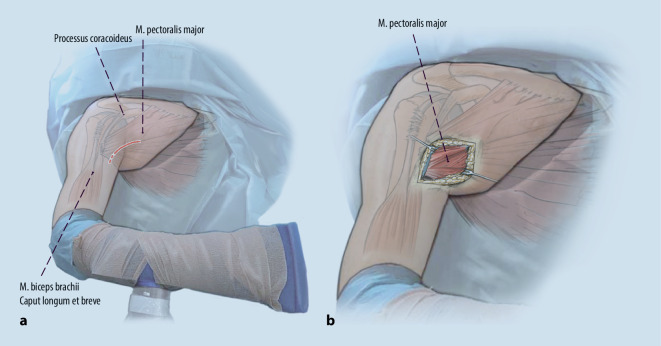

**Figure 5:**
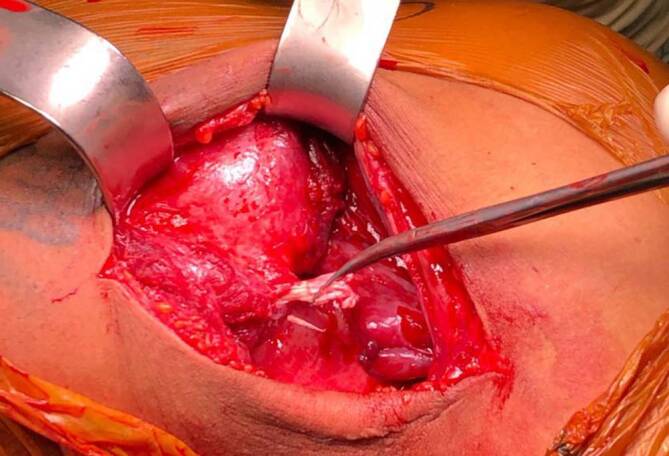

**Figure 6:**
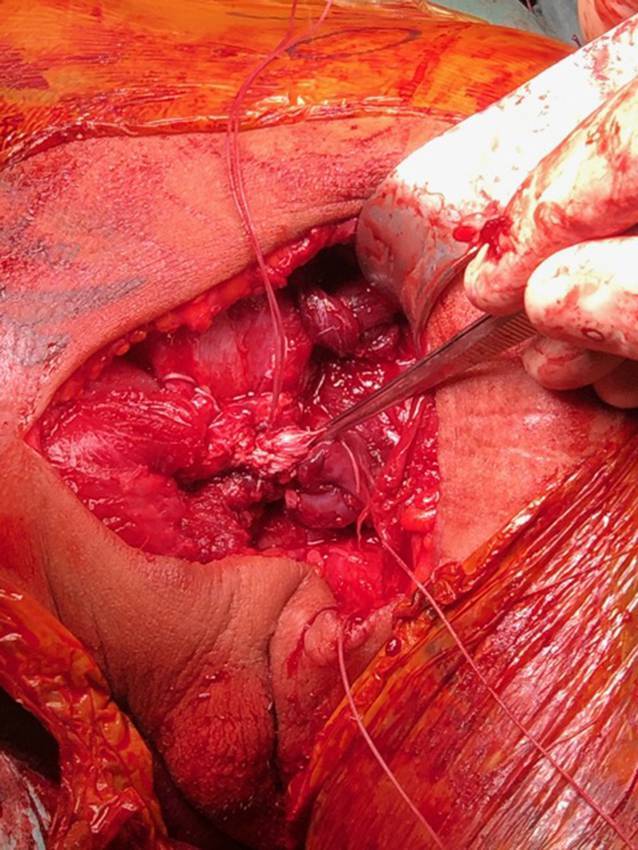

**Figure 7:**
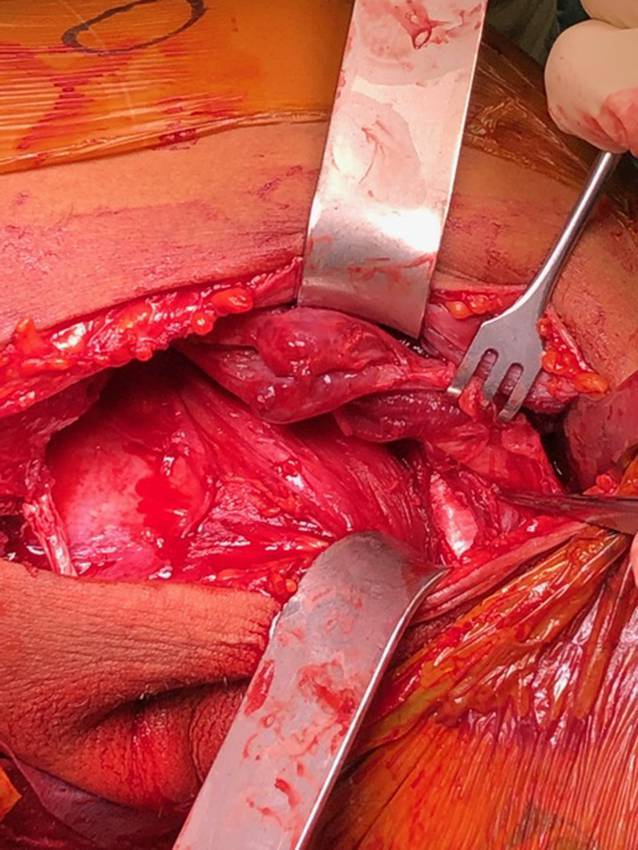

**Figure 8:**
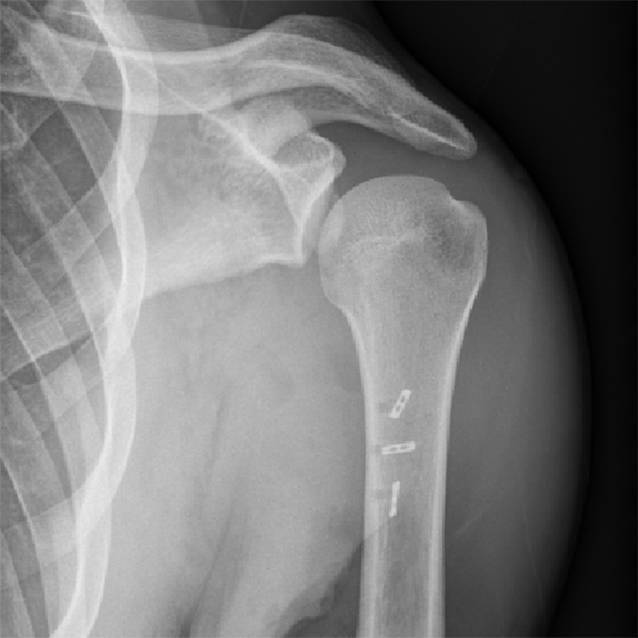

Über einen bogenförmigen Hautschnitt in der vorderen Axillarfalte (Abb. 4) wurden nach Präparation des Subkutangewebes zunächst die sternokostalen und klavikulären Muskelanteile dargestellt, die weit nach medial retrahiert waren. Wie aus der MRT-Untersuchung bekannt, konnte die Ruptur am muskulotendinösen Übergang bestätigt werden (Abb. 5). Die kaudalen Sehnenzügel der Pars abdominalis inserierten noch regelhaft. Zur humeralseitigen Refixation wurden insgesamt 3 Flip-Plättchen (Pec-Button, Arthrex, Naples, FL, USA) intramedullär platziert. Die intramedulläre Lage der Buttons wurde mittels Bildverstärker (BV) kontrolliert. Die Buttons werden vor der Implantation mit einem nichtresorbierbaren Tape-Faden (FiberTape®, Arthrex, Naples, FL, USA) geladen. Im eigenen Vorgehen erfolgte die Reinsertion extraanatomisch medial der langen Bizepssehne im Bereich der Crista tuberculi minoris. Nach Meinung der Autoren bietet die medialisierte Reinsertion (im Gegensatz zur anatomischen Insertion lateral der langen Bizepssehne) den Vorteil, ein sekundäres Impingement der langen Bizepssehne durch potenzielle Vernarbungen zu verhindern sowie einem Außenrotationsdefizit durch verkürzte Sehnenzügel vorzubeugen [8]. Der muskulotendinöse Übergang der Pars sternocostalis wurde mit dem kranialsten Tape-Faden mit einer auf- und absteigenden Krackow-Naht armiert. Anschließend folgte die Armierung der Pars clavicularis in identischer Weise mittels des kaudalen Tape-Fadens (Abb. 6). Über Zug an den jeweiligen freien Tape-Enden erfolgte die humeralseitige Reposition in Neutralstellung des Armes. Anschließend wurde der dritte Tape-Faden in die Pars sternocostalis eingeflochten und somit der gesamte Muskel unter gleichmäßigem Zug aller Tapes epiossär am Humerus reinseriert (Abb. 7). Der Wundverschluss erfolgte über einer eingelegten Redon-Drainage. Das postoperativ angefertigte Röntgenbild bestätigte erneut die korrekte intramedulläre Lage der Pec-Buttons (Abb. 8).

Postoperativ wurde die linke Schulter mittels Schulterabduktionskissen in 15°-Abduktion für 6 Wochen ruhiggestellt. Für diesen Zeitraum wurden eine passive Abduktion und Anteversion bis 30° und Außenrotation bis 0° freigegeben. Die aktive Innenrotation wurde strikt limitiert.

**Figure 9:**
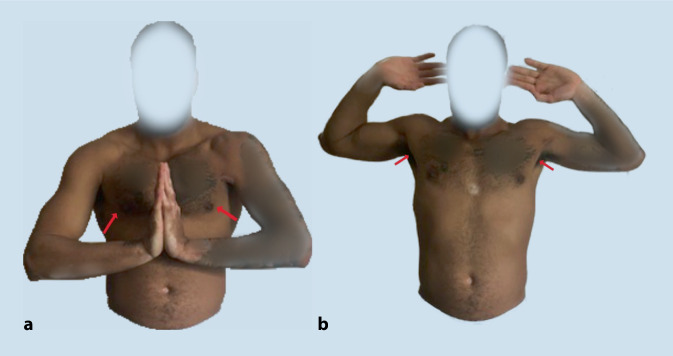

Im Anschluss an die klinische Kontrolle 6 Wochen postoperativ konnte bei regulärem Heilungsverlauf die freie aktiv-assistierte Beweglichkeit im schmerzfreien Intervall sukzessive gesteigert werden. Zwei Monate postoperativ konnte bereits das volle Bewegungsausmaß wieder erreicht werden (Abb. 9). Drei Monate nach erfolgter Refixation konnte der Patient für das Basketballtraining freigegeben werden.

## Diskussion

Patienten, die eine PMR erleiden, berichten häufig von einem schnalzenden Geräusch während der Verletzung. In einzelnen Fällen kann jedoch entlang der vorderen Brustwand oder häufiger im Bereich der humeralseitigen Insertion ein Hämatom auftreten (dann meist schon als älteres, verfärbtes Hämatom bei verzögerter Vorstellung). In der körperlichen Untersuchung imponieren, neben einer lokalen Schmerzsymptomatik, eine Innenrotationsschwäche (ggf. mit positivem „prayer sign”) und nicht selten ein pathologisches Konturzeichen [8, 10].

In der 1980 von Tietjen publizierten Klassifikation werden PMR nach dem Ausmaß der Ruptur (Typ I: Muskelzerrung, Typ II: Partialruptur, Typ III: Komplettruptur) und Komplettrupturen zusätzlich nach der Lokalisation (A: Muskelansatz, B: Muskelbauch, C: muskulotendinöser Übergang, D: Sehneninsertion) eingeteilt, wobei ausschließlich Typ-III-Rupturen chirurgisch adressiert werden sollten [9]. Diese rein anatomische Klassifikation wurde 2012 von ElMaraghy et al. [7] erweitert. Sie umfasst zusätzlich das Ausmaß der Defektbreite und -tiefe sowie die Einteilung in akute und chronische Rupturen [7]. Roller et al. publizierten 2006 eine weitere Klassifikation zur Einteilung von PMR mit 3 verschiedenen Rupturtypen, welche 2010 durch Ritsch modifiziert wurde (Typ 1: tendinöse Ruptur, Typ 2: muskulotendinöse Ruptur, Typ 3: muskuläre Ruptur) [11, 12]. Anhand der Klassifikation von Roller et al. [11] wird die operative Versorgung der hier vorgestellten Typ-2-Ruptur je nach intraoperativem Befund durch eine End-zu-End-Naht oder knöcherne Refixation empfohlen.

In eine aktuelle prospektive randomisierte Studie wurden 60 Patienten mit kompletter PMR eingeschlossen (31 operativ und 29 konservativ behandelt). Für die operativ behandelten Patienten konnte ein signifikant besseres funktionelles Ergebnis belegt werden, wobei lediglich ein Patient mit einer PMR am muskulotendinösen Übergang eingeschlossen wurde [13].

Auch Bodendorfer et al. [14] berichteten in ihrer Metaanalyse von 23 Studien mit insgesamt 664 Fällen von einem signifikant besseren Outcome operativ versorgter PMR im Vergleich zur konservativen Therapie in Bezug auf das funktionelle Ergebnis (23,3 % besser in den Bak-Kriterien), die isometrische und isokinetische Kraft sowie das kosmetische Resultat. Trotz guter funktioneller Ergebnisse war die operative Versorgung von PMR mit einer Komplikationsrate von 14 % verbunden, wobei die häufigsten Komplikationen die Reruptur und persistierender Schmerz waren. Allerdings wurde jedoch nicht zwischen den einzelnen Rupturformen unterschieden [14].

Eine weitere Untersuchung zeigte, dass das funktionelle Outcome und die kosmetische Zufriedenheit signifikant besser bzw. höher für akut versorgte PMR als für chronische Rupturen (älter als 6 Wochen) waren [15, 16].

Da es sich bei der Mehrzahl der Patienten um Athleten oder Patienten mit hohem funktionellen Anspruch handelt, ist prinzipiell eine Empfehlung für eine zeitnahe operative Rekonstruktion auszusprechen [13]. Man kann also ableiten, dass die operative Therapie von PMR, idealerweise im akuten Stadium, bei tendinösen Avulsionen der konservativen Therapie überlegen ist. Bei intramuskulären Rupturen des PM, einer sehr seltenen Entität, konnte im Rahmen von Fallberichten gezeigt werden, dass eine direkte Naht zu guten Ergebnissen führen kann [17, 18].

Schwieriger ist die Therapieentscheidung (konservativ vs. operativ) jedoch bei Rissen im muskulotendinösen Übergang. Eine Studie von Bak et al. [6] hat sich zwar mit der Einteilung der Ruptur nach Tietjen [9] befasst, konnte aber, am ehesten aufgrund von kleinen Gruppen (z. B. 21 Patienten mit einer muskulotendinösen Ruptur) keinen signifikanten Unterschied in den Ergebnissen nach Rupturen in den verschiedenen Anteilen zeigen [6]. Da aber die Ergebnisse nach konservativer Therapie allgemein deutlich unterlegen sind, sollte auch hier, wenn möglich, eine operative Refixation erfolgen [14]. Zur Augmentation der fehlenden stichfesten Sehnenstruktur wurde daher bei muskulotendinösen Rupturen auch die Einflechtung von einem Sehnen-Allografts durch den Muskel und die robuste dorsale Faszie, mit guten klinischen Ergebnissen, beschrieben [19].

Alternativen zu der beschriebenen Refixationsmethode mittels intramedullärer Pec-Buttons sind die transossäre Naht bzw. direkte Naht, die Ankerrefixation oder die Verwendung von Schrauben mit Beilagscheiben [16]. Rabuck et al. simulierten in einer biomechanischen Studie die Belastbarkeit der transossären Naht, intramedullärer Kortikalis-Buttons und der Ankernaht während des Bankdrückens und konnten feststellen, dass die transossäre Naht sich als signifikant belastbarer als die Ankernaht herausstellte. Der Unterschied bei der Verwendung von Kortikalis-Buttons im Vergleich zur transossären Naht war nicht signifikant [20]. Hart et al. und Sherman et al. stellten in ihren Untersuchungen keine signifikanten Unterschiede im Hinblick auf die Belastbarkeit der verschiedenen Refixationsmethoden fest [21, 22]. Auch in der Metaanalyse von Bodendorfer et al., welche 122 Refixationen mittels transossärer Nähte, 78 Kortikalis-Button-Refixationen, 74 Refixationen mit Ankernähten-, 12 Refixationen durch Schrauben mit Beilagscheiben und 26 direkte Nähte einschloss, konnten keine signifikanten Unterschiede in Bezug auf funktionelle Scores (SANE, ASES, DASH) sowie die weiteren Parameter (Bewegungsumfang, Kraft, Schmerzfreiheit, „return to activity“, Komplikationsrate, Zufriedenheit mit der physischen Erscheinung und der allgemeinen Zufriedenheit mit dem Ergebnis) festgestellt werden [16].

Die häufigsten beschriebenen Komplikationen bei akuter Versorgung waren neurologische Schäden (ca. 5 %) im Rahmen eines vorübergehenden Defizits des Fasciculus medialis des Plexus brachialis [23], Rerupturen (ca. 3 %) und postoperativ aufgetretene Hämatome (ca. 3 %) [16]. Etwa 97 % der Patienten mit operativ versorgter PMR erreichten den vollen Bewegungsumfang zurück [14].

Im vorliegenden Fall entschieden wir uns für eine intramedulläre Pec-Button-Refixation des PM. Diese Technik bietet einerseits eine hohe Ausrisskraft und andererseits den Vorteil kleiner Bohrlochdurchmesser zur Minimierung eines möglichen Frakturrisikos am Humerusschaft [20, 21, 24]. In der eigenen Arbeitsgruppe konnten bereits exzellente biomechanische und klinische Ergebnisse bei der Versorgung von Bizepssehnenrupturen bzw. -tenodesen für diese Refixationsmethode gezeigt werden [25–28]. Die wahlweise Verwendung von Tape-Fäden ermöglicht es, die Auflagefläche der Naht zu vergrößern und somit das potenzielle Einschneiden in intaktes Gewebe verringern.

Die einzelnen Refixationsmethoden haben in ihrer Anwendung sowohl Vor- als auch Nachteile. Der Vorteil der transossären Naht liegt darin, dass eine große Kontaktfläche zwischen Sehne und Knochen geschaffen wird. Allerdings wird dabei eine nicht unerhebliche Strecke des Sehnenendes im Knochen versenkt (intraossäre Refixation) und die Sehne dadurch verkürzt, was v. a. bei Rupturen mit starker Retraktion eine Refixation erschweren und u. U. eine eingeschränkte ARO bedingen kann. Bei der Nutzung transossärer Nähte ist – im Vergleich zur Anwendung von Ankernähten oder Buttons – außerdem eine größere Dissektion des umgebenden Gewebes erforderlich, was das Risiko für iatrogene Schäden bei nah angrenzenden Strukturen wie den Sehnen des M. latissimus dorsi und des M. teres major erhöhen kann [21, 22, 29]. Transossäre Nähte bieten jedoch den Vorteil geringerer Materialkosten [21]. Die direkte Naht findet v. a. bei der Versorgung von inkompletten Rupturen mit intakter distaler Restsehne Anwendung [30]. Im beschriebenen Fall war eine direkte Naht aufgrund des Ausmaßes der Ruptur mit insuffizientem Sehnenstumpf nicht möglich. Bei ähnlichen biomechanischen Ergebnissen wird die Wahl der Refixationsmethode vorrangig durch die Präferenz des Chirurgen, die Rupturform und die Implantatkosten beeinflusst.

Abschließend kann zusammengefasst werden, dass eine chirurgische Intervention bei PMR insbesondere Patienten mit hohem funktionellen Anspruch empfohlen werden sollte, da die funktionellen Ergebnisse einer chirurgischen Versorgung der konservativen Therapie deutlich überlegen sind.

Nach Ansicht der Autoren kann die Nutzung intramedullärer Buttons empfohlen werden, da sie eine zuverlässige Fixation bei geringem Frakturrisiko gewährleisten.

## Fazit für die Praxis

- Die Ruptur des M. pectoralis major ist eine Verletzung, die maßgeblich bei sportlichen Aktivitäten – v. a. beim Kraftsport (Bankdrücken) – auftritt.
- Das operative Management ist – auch bei Rupturen im muskulotendinösen Übergang – der konservativen Therapie insbesondere bei Patienten mit hohem funktionellen Anspruch vorzuziehen.
- Die Verwendung intramedullärer Buttons hat sich zur Versorgung von Sehnenrupturen bereits klinisch bewährt.
